# The criterion for dividing the surrounding rock EDZs of underground caverns based on the energy dissipation degree

**DOI:** 10.1371/journal.pone.0288324

**Published:** 2023-07-28

**Authors:** Xianliang Wang, Jianhai Zhang, Li Qian, Tianzhi Yao, Zuguo Mo, Jianhua He, Ru Zhang, Changgui Zhao, Zanbo Qiao

**Affiliations:** 1 State Key Laboratory of Hydraulics and Mountain River Engineering, College of Water Resources and Hydropower, Sichuan University, Chengdu, China; 2 Sichuan Metallurgical Geological Survey and Design Group Corporation Limited, Chengdu, China; 3 Power China Chengdu Engineering Corporation Limited, Chengdu, China; Xi’an University of Science and Technology, CHINA

## Abstract

An energy calculation parameter named the energy dissipation degree (*R*_*Ud*_) is introduced based on the analysis of the energy dissipation mechanism and energy evolution characteristics during conventional triaxial tests of the granite of Shuangjiangkou. The deviatoric stress‒strain curve of rock can be divided into five stages using four stress thresholds (crack closure stress *σ*_*cc*_, crack initiation stress *σ*_*ci*_, damage stress *σ*_*cd*_ and peak stress *σ*_*p*_), which also correspond to the four *R*_*Ud*_ thresholds (*R*_*Udc*_, *R*_*Udi*_, *R*_*Udd*_ and *R*_*Udp*_) on the energy dissipation degree–strain curve. A given stress threshold increases with increasing confining pressure; however, a given *R*_*Ud*_ threshold is basically stable under different confining pressures. Then, a new criterion for dividing the excavation damaged zones (EDZs) in the rock surrounding underground caverns based on the monotonically increasing characteristics of the energy dissipation degree‒axial strain relationship curve is proposed, and it allows for the classification of the surrounding rock into five types of zones through quantitative analysis of the *R*_*Ud*_ thresholds. Based on the criterion for dividing the EDZs of the surrounding rock mass of the underground cavern, the EDZs of the surrounding rocks of the underground cavern group of the Shuangjiangkou Hydropower Station are analyzed. The distribution characteristics of the EDZs of the rock surrounding underground caverns obtained by numerical simulation calculations based on *R*_*Ud*_ are basically the same as those obtained by in situ elastic wave tests. However, the *R*_*Ud*_-based method for classifying the EDZs of the surrounding rock has the obvious advantage of being able to probe the boundaries of the undamaged zone (UDZ) of the surrounding rock more explicitly, while the method based on wave velocity testing is not sufficiently explicit. The damage zoning of the surrounding rock based on *R*_*Ud*_ can provide support design advice for the excavation of the surrounding rock, such as the support method, the length of the free section and anchor section of the prestressing anchor, etc.

## 1. Introduction

During the construction of underground projects, rock excavation and the protection of surrounding rock mass stability are conflicting but necessary challenges. When using static and dynamic rock breakage methods (soundless cracking agents, tunnel boring machine, gas blasting, rock blasting, etc.) to excavate rock, the excavation and unloading process will disrupt the ground stress balance and cause redistribution of stress in the surrounding rock [1–4]. The energy stored in the surrounding rock is gradually dissipated, while geological, thermal, mechanical, hydraulic, chemical and acoustic characterization of the surrounding rock are affected [5–7]. The damage and destruction of the surrounding rock inevitably occurs, resulting in excavation-disturbed zones or excavation-damaged zones (EDZs) [8]. The temporal and spatial evolution of EDZs are characterized by the gradual development of the surrounding rock from a relatively intact state (in situ stress) to a fully adjusted state (new stress equilibrium state after excavation) under the influence of excavation [9]. The formation and expansion of EDZs are the root cause of deformation and the damage of the underground cavity envelope, causing challenges related to stability and seepage, thus impairing the performance and function of the surrounding rock mass [10]. Therefore, it is critical to select reasonable and practical methods to accurately determine the formation mechanism, change trend, and the range of the EDZs of the surrounding rock to provide support for the design of underground cavern support.

To better conduct research on EDZs, many countries or regions have set up specialized underground research laboratories (URLs), such as the URL for the Lac du Bonnet granite in Canada [8, 9], the Mont Terri Rock Laboratory for the Opalinus Clay in Switzerland [10, 11], the Äspö Hard Rock Laboratory for granite in Sweden [12], the URL for the Méraillet crystalline rock in the French Alps [13], the Tournemire URL in Francev [14], the Meuse/Haute-Marne URL in France [15–17], the High-Activity Disposal Experimental Site in Belgium [18], the Tono mine in Japan [19], the Horonobe URL in Japan [20, 21], the KAERI Underground Research Tunnel in Korea [22] and the Jinping URL in China [23, 24].

To quantitatively determine the range and variation pattern of EDZs, scholars have conducted studies based on various instruments to record relevant data, such as displacement and strain measurement [19, 25], wave velocity testing [24, 26], fluid permeation testing [21, 27, 28], gas permeation testing [5, 29], resistivity testing [12, 30, 31], acoustic emission and microseismic event monitoring [32–34], borehole core analysis [13, 35], chemical test methods [36, 37], seismic wave testing [38–41], borehole camera technology [42, 43], drill hole panoramic digital imaging technology [24], filling resin observation [17, 44], and physical model tests [33]. It has been shown that the characteristics of EDZs vary with the rock properties, in situ stress field, geological environment, excavation method, and cavity section geometry, and the results obtained from different research methods vary. The relevant data recorded by the instruments reflect the unique thermal, mechanical, hydrodynamic, chemical, and acoustic characteristics of EDZs, which can be used to gain insight into the mechanisms of EDZ formation, and to determine the extent and variation patterns of EDZs. Nevertheless, owing to topographical and financial constraints, among other factors, the amount of data recorded by instrumentation is limited, challenging the assessment of the extent and degree of EDZs. Numerical simulation methods have also been widely used for the study of EDZs [45–48]. In addition, some scholars use reliability analysis tools to estimate the probability of damage zone generation for the optimal design of support measures [49]. These methods for generating quantitative results are often used in combination to improve the accuracy of the methodology of the study [12, 14, 22, 25, 48].

Based on the analysis of the energy variation in the Shuangjiangkou granite via conventional triaxial tests, the energy calculation parameter *R*_*Ud*_ is introduced in the paper. Then, the criterion for dividing the EDZs in the rock surrounding underground caverns based on *R*_*Ud*_, which considers the effects of both confining pressure and strain, is proposed. The surrounding rock EDZs of the underground caverns of the Shuangjiangkou Hydropower Station are classified by applying the criterion through numerical simulation. In the 0+070.00 m section, eight in situ wave velocity test boreholes have been arranged in the main powerhouse, the main transformer chamber and the tailwater surge chamber, and the EDZs of the surrounding rock mass are classified according to the variation in wave velocity with the depth of the borehole. The EDZs based on elasticity wave testing data from in situ tests are compared with the results obtained from numerical simulations. Analyzing the surrounding rock’s overall stability through zoning can provide a reference for the design of support.

## 2. Method and experimental results

### 2.1 Conventional triaxial tests

**Fig 1:**
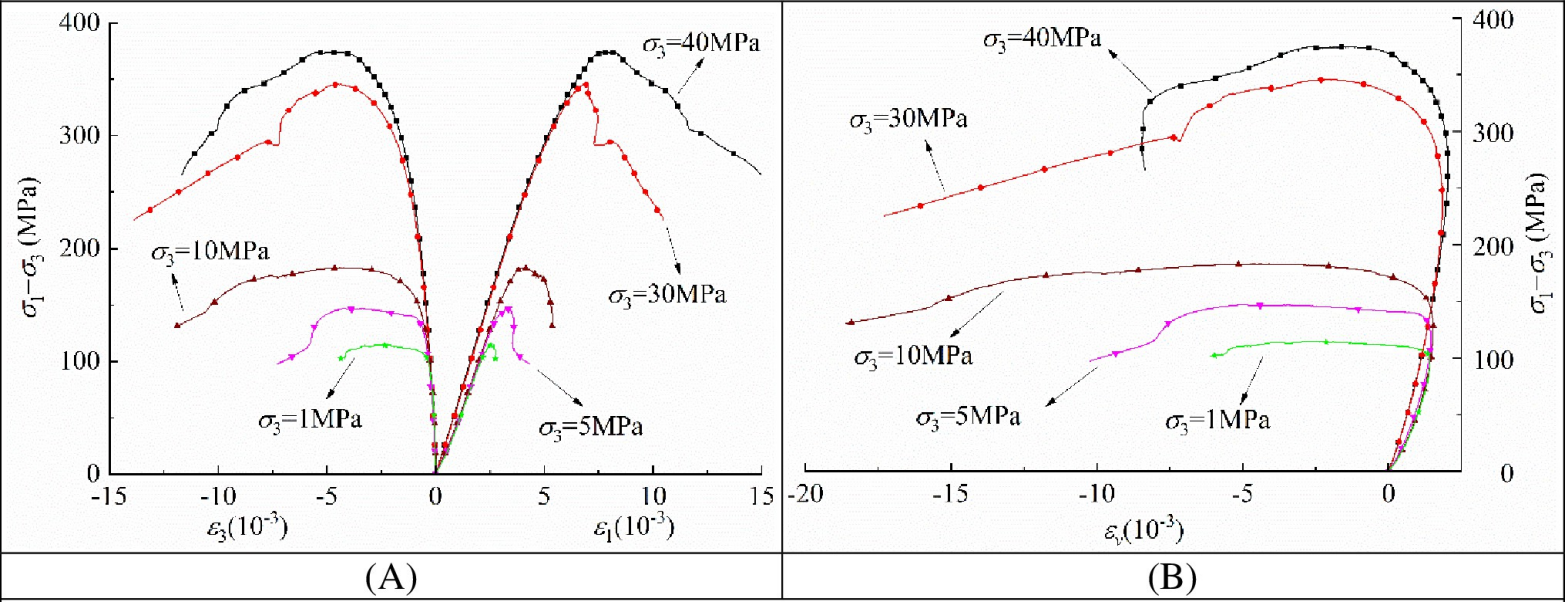
The conventional triaxial deviatoric stress‒strain curves [50].

Shuangjiangkou Hydropower Station is located in the upper reaches of the Dadu River in China, and the rock in which the underground powerhouse engineering is located consists of granite. The deviatoric stress‒strain relationship of granite under different envelope pressures is obtained by conventional triaxial tests, shown in Fig 1 [50].

### 2.2 The energy dissipation degree *R*_*Ud*_

According to the principle of thermodynamics, the total energy received by granite samples during tests can be calculated by Eq (1) [51, 52].

$$U=U_{l}+U_{e}+U_{d},$$ (1)

where *U*, *U*_*l*_, *U*_*e*_ and *U*_*d*_ represent the total absorbed energy density, freezing energy density, elastic strain energy density and the dissipated energy density, respectively.

Since the amount of freezing energy is remarkably small compared to the elastic strain energy and the dissipation energy [52], an approximation is taken in the energy calculation, ignoring the effect of freezing energy on the deformation damage of the rock sample, so Eq (1) is simplified to Eq (2),

$$U=U_{e}+U_{d}.$$ (2)

Eq (3) can be used to calculate the total absorbed energy density,

$$U=U_{0}+U_{1}+U_{3},$$ (3)

where *U*_0_ is the energy density caused by hydrostatic loading, *U*_1_ is the energy density owing to axial compression by *σ*_1_ after hydrostatic pressure, and *U*_3_ is the energy density owing to lateral compression by *σ*_3_ after hydrostatic pressure.

$$U_{0}=\frac{3(1-2\mu )}{2E}\sigma_{3}^{2},$$ (4)

$$U_{1}=\int \sigma_{1}d\varepsilon_{1}=\sum_{i=0}^{n}\frac{1}{2}(\varepsilon_{1(i+1)}-\varepsilon_{1i})(\sigma_{1(i+1)}+\sigma_{1i}),$$ (5)

$$U_{3}=2\int \sigma_{3}d\varepsilon_{3}=\sum_{i=0}^{n}\left(\varepsilon_{3(i+1)}-\varepsilon_{3i})(\sigma_{3(i+1)}+\sigma_{3i}\right),$$ (6)

where *μ*, *E*, *σ*_1*i*_, *ε*_1*i*_, *σ*_3*i*_, and *ε*_3*i*_ are Poisson’s ratio, elastic modulus, axial stress, axial strain, lateral stress, and lateral strain at point *i* on the stress–strain curve, respectively.

$$U_{e}=\frac{1}{2}(\sigma_{1}\varepsilon_{1}^{e}+2\sigma_{3}\varepsilon_{3}^{e})=\frac{1}{2E}[\sigma_{1}^{2}+2(1-\mu )\sigma_{3}^{2}-4\mu \sigma_{1}\sigma_{3}],$$ (7)

the dissipated energy can be obtained by Eqs (2)–(7) [50, 51].

The rock damage evolution process will lead to energy dissipation, and the study of the damage evolution characteristics of rocks from the perspective of energy dissipation can fundamentally reflect the deformation and damage characteristics of rocks. To better study the energy evolution of granite during the damage process of the Shuangjiangkou Hydropower Station, an energy calculation parameter named the energy dissipation degree (*R*_*Ud*_) is introduced. *R*_*Ud*_ is defined as the ratio of the dissipation energy during the evolution of rock damage to the dissipation energy at the peak stress, and *R*_*Ud*_ represents the degree of dissipation energy change during the damage of the rock, which can be obtained by Eq (8).


$$R_{Ud}=\frac{U_{d}}{U_{d}^{p}},$$ (8)

**Fig 2:**
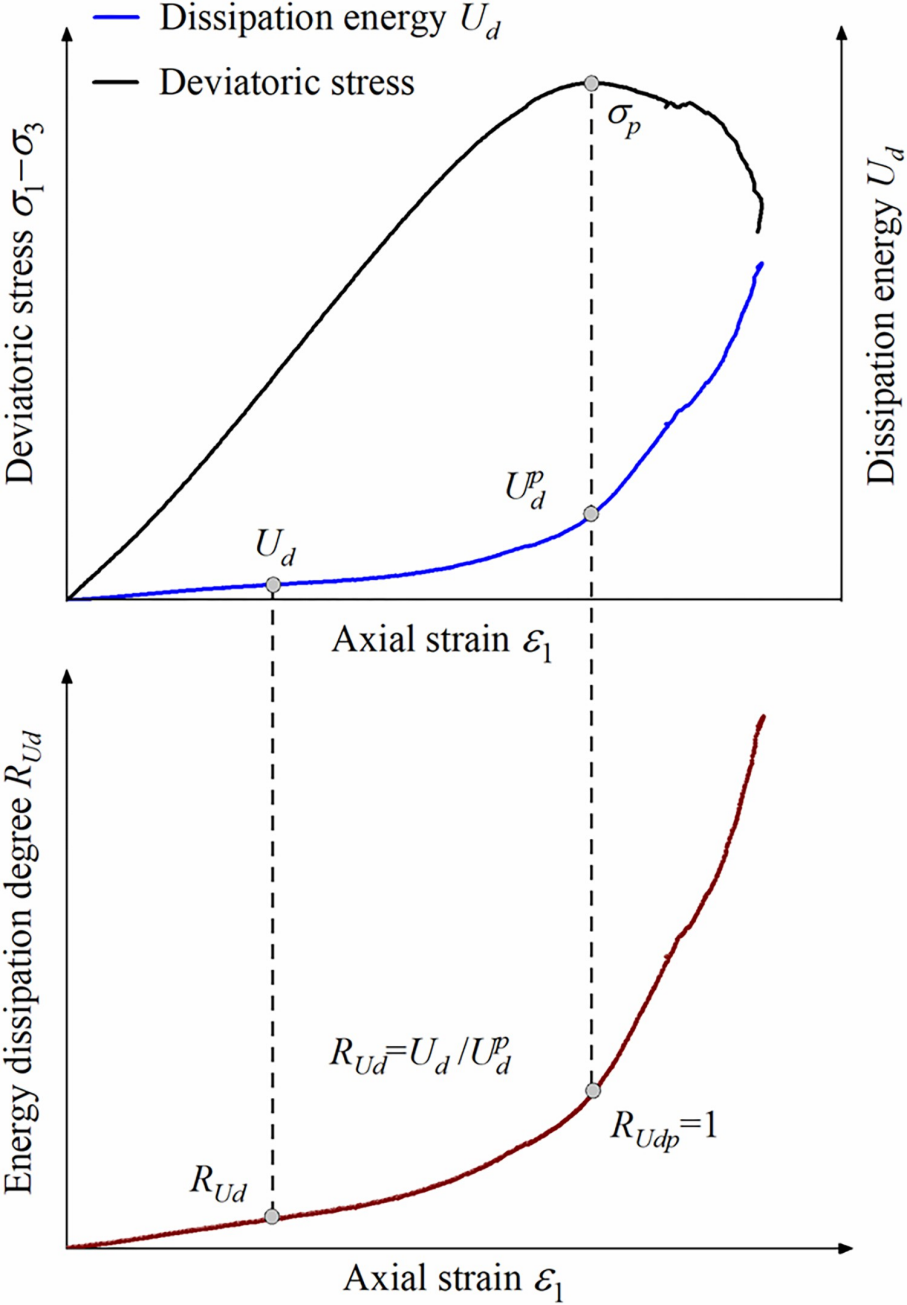
Schematic diagram of the definition of *R*_*Ud*_.

Fig 2 shows the definition of the energy dissipation degree *R*_*Ud*_. From this figure, it can be seen that when *U d* <*Up d*, *R*_*Ud*_ <1; when *U d* = *Up d*, *R*_*Ud*_ = 1; and *U d* >*Up d*, *R*_*Ud*_ >1.

**Fig 3:**
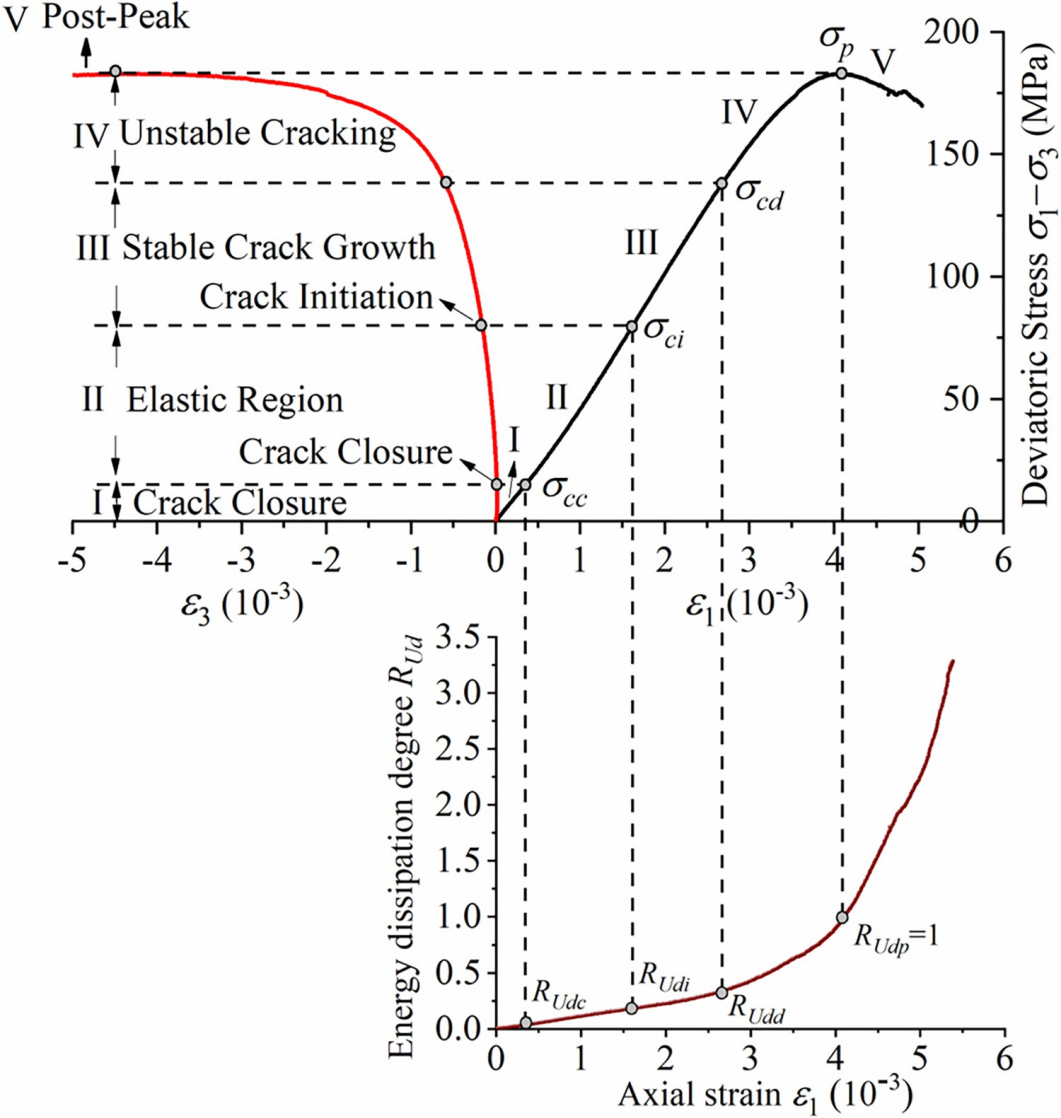
Method of determining *R*_*Ud*_ thresholds.

A large number of studies [53–55] have shown that, the deviatoric stress‒strain curve of rock can be divided into five stages using four stress thresholds: crack closure stress (*σ*_*cc*_), crack initiation stress (*σ*_*ci*_), damage stress (*σ*_*cd*_) and peak stress (*σ*_*p*_). The four stress thresholds correspond to the four *R*_*Ud*_ thresholds (*R*_*Udc*_, *R*_*Udi*_, *R*_*Udd*_ and *R*_*Udp*_) on the energy dissipation degree-strain curve, as shown in Fig 3.

**Fig 4:**
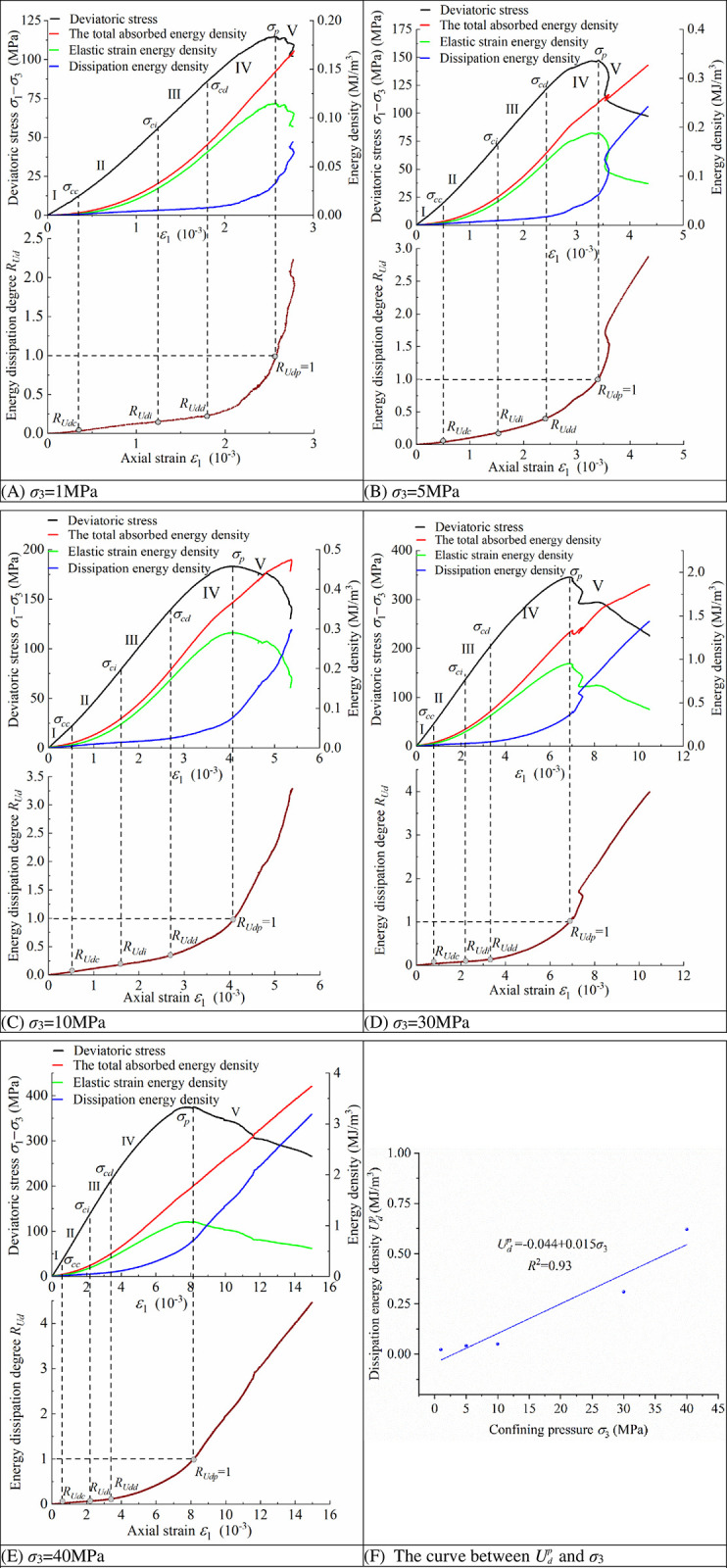
Threshold values for energy dissipation degree at different confining pressures and the curve between *Up d* and *σ*_3_.

The determination of the energy dissipation degree thresholds is shown in Table 1 and Fig 4.

**Table 1:** The threshold values of energy dissipation degree *R*_*Ud*_ and dissipation energy density *Up d*.

*σ*_3_ (MPa)	*σ*_*cc*_ (MPa)	*R*_*Udc*_		*σ*_*ci*_ (MPa)	*R*_*Udi*_		*σ*_*cd*_ (MPa)	*R*_*Udd*_		*σ*_*p*_ (MPa)	*R*_*Udp*_	*Up d* (MJ/m^3^)
1	9.10	0.020	0.02	56.75	0.151	0.17	86.66	0.326	0.34	114.76	1	0.023
5	10.35	0.016		71.65	0.178		120.67	0.362		147.12		0.042
10	15.19	0.026		79.80	0.181		138.69	0.342		183.04		0.051
30	27.07	0.022		134.02	0.153		202.05	0.314		345.56		0.310
40	32.71	0.018		144.92	0.169		213.82	0.306		374.17		0.621

The stress threshold at different confining pressures becomes larger with increasing confining pressure, while the corresponding threshold of energy dissipation degree *R*_*Ud*_ varies in a smaller interval at different confining pressures. The variation interval of *R*_*Udc*_ is [0.016, 0.026], *R*_*Udi*_ [0.151, 0.181] and *R*_*Udd*_ [0.306, 0.362]. It can be seen that the same threshold has good consistency under different confining pressures, therefore, using the method of statistically calculating the average of the thresholds at different confining pressures, *R*_*Udc*_ can be taken as 0.02, *R*_*Udi*_ as 0.17, *R*_*Udd*_ as 0.34, while *R*_*Udp*_ is 1 as defined by Eq (8).

## 3. The criterion for dividing EDZs based on *R*_*Ud*_

### 3.1 Definition of the criterion for dividing EDZs based on *R*_*Ud*_

In analyzing the EDZs of the surrounding rock encountered in underground cavities, stress, deformation, and plastic zone variation characteristics are generally taken as the foci of research. In contrast, less research has been conducted to analyze EDZs based on energy dissipation characteristics [47, 56]. The damage evolution characteristics of rocks are studied from the perspective of energy dissipation, which can reflect the deformation process of rocks.

**Fig 5:**
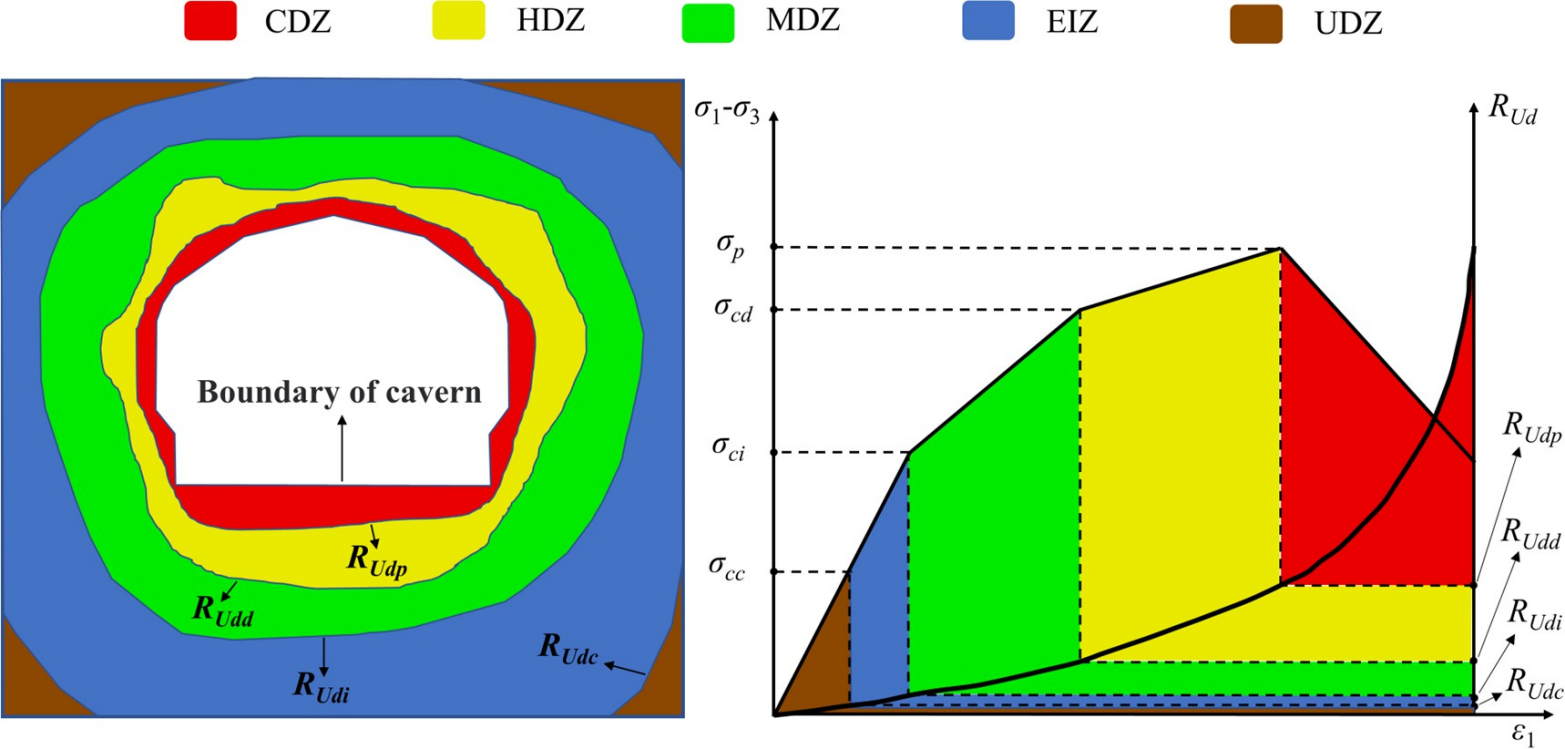
Schematic diagram of the criterion for dividing EDZs based on *R*_*Ud*_.

In underground cavity excavation, the initial ground stress is unbalanced owing to the excavation activities, which redistribute the stress of the surrounding rock. In general, the stress concentration will occur tangential to the cavern wall, whereas stress relaxation will occur along the cavern wall’s radial direction, and even tensile stress will appear. Such unfavorable stress conditions result in varying degrees of rock damage, and the destruction around the cavity chamber and the surrounding rock can be classified as five circles according to the distance from the cavity chamber, from near to far (Fig 5).

The construction-damaged zone (CDZ) is generally located immediately around the underground cavity chamber, where the surrounding rock’s damage and destruction is serious, while numerous macroscopic cracks or fracture surfaces are formed, and the surrounding rock loses its bearing capacity. The surrounding rock in this part can be supported by initial protection with shotcrete immediately after excavation, and then supported by system anchors with reinforcement mesh and poured concrete lining. The pressure generated in this area is loose ground pressure and should be used as the basis for the design of the pressure to which the support is subjected. The highly damaged zone (HDZ) is generally located in the second circle around the underground cavity chamber, in which the surrounding rock’s damage is serious, and additional macrocracks or rupture surfaces occur, seriously weakening the surrounding rock’s bearing capacity. The pressure generated in the area where CDZ and HDZ are located is loose ground pressure and should be used as the basis for the design of the pressure to which the support is subjected. The mildly damaged zone (MDZ) is located in the third circle around the underground cavity, in which the surrounding rock’s damage is slight and there are fewer macrocracks or rupture surfaces; thus, the surrounding rock has a certain bearing capacity. The pressure generated in the area where MDZ is located can be used as the design basis for the pressure on the support, and it should be used as the key monitoring part for the stress and displacement of the surrounding rock, and then the working condition of the surrounding rock and support can be monitored in real time. The excavation-influenced zone (EIZ) is located in the fourth circle around the underground cavity, where the surrounding rock is influenced, there are fewer cracks, and the surrounding rock is of better quality, with fewer cracks and stronger bearing capacity. The area where the EIZ is located can be used as a key monitoring site for the surrounding rock stress and displacement, and then the working condition of the surrounding rock and support can be monitored in real time. The undamaged zone (UDZ) includes the very slightly disturbed area and the original rock area. When pre-stressed anchors are used for support design, the area in which CDZ, HDZ, MDZ and EIZ are located should be designed as the free section of anchors, while the area in which UDZ is located can be designed as the anchored section of anchors.

Based on conventional triaxial tests, the deviatoric stress‒axial strain relationship curves and the energy dissipation degree–axial strain relationship curves are used as the basis for dividing the EDZs of the surrounding rock. This is the criterion proposed for identifying the EDZs in the rock surrounding underground caverns based on the energy dissipation degree *R*_*Ud*_, as shown in Fig 5. The criterion uses four energy dissipation degree thresholds (*R*_*Udc*_, *R*_*Udi*_, *R*_*Udd*_ and *R*_*Udp*_) to classify the surrounding rock mass into five categories: CDZ, HDZ, MDZ, EIZ and UDZ.

### 3.2 Theoretical basis of the criterion for dividing EDZs based on *R*_*Ud*_

It is well known that the deviatoric stress‒axial strain relationship curve of rock is based on a specific confining pressure. Thus, the resulting energy dissipation degree *R*_*Ud*_–axial strain relationship curve is also based on a specific surrounding pressure, while the surrounding rock’s different position corresponds to different stress states. Therefore, the energy dissipation degree *R*_*Ud*_–axial strain relationship curve at a specific surrounding pressure should correspond to the energy dissipation state at any location in the surrounding rock.

**Fig 6:**
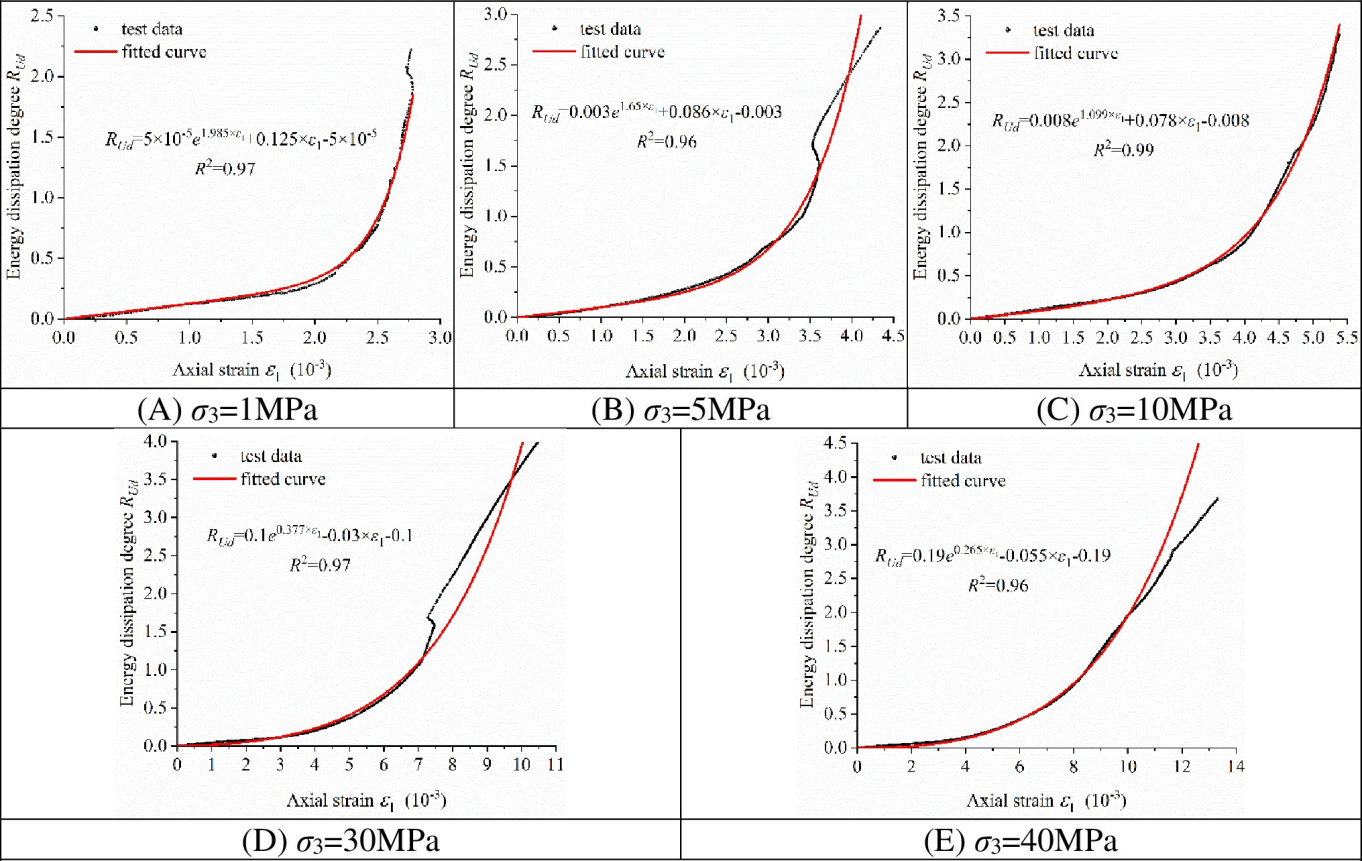
Fitting of the energy dissipation degree–axial strain relationship curves at different confining pressures.

Fig 6 shows five curves of the relationship between the energy dissipation degree *R*_*Ud*_ and the axial strain of granite in the Shuangjiangkou Hydropower Station at different confining pressures obtained by conventional triaxial tests. Through curve fitting, the five curves can be expressed with a simple monotonic increasing function, as shown in Eq (9).


$$R_{Ud}=A_{1}\times e^{A_{2}\varepsilon_{1}}+A_{3}\times \varepsilon_{1}+A_{4},$$ (9)

The relevant fitting coefficients *A*_1_, *A*_2_, *A*_3_ and *A*_4_ and their values are shown in Table 2.

**Table 2:** The relevant fitting coefficients.

*σ*_3_(MPa)	*A*_1_	*A*_2_	*A*_3_	*A*_4_
1	5×10^−5^	1.985	0.125	-5×10^−5^
5	0.003	1.650	0.086	-0.003
10	0.008	1.099	0.078	-0.008
30	0.100	0.377	-0.030	-0.100
40	0.190	0.265	-0.055	-0.190

Fig 6 shows that all the curves are well fitted, and the correlation coefficients *R*^2^ reach above 0.96.

**Fig 7:**
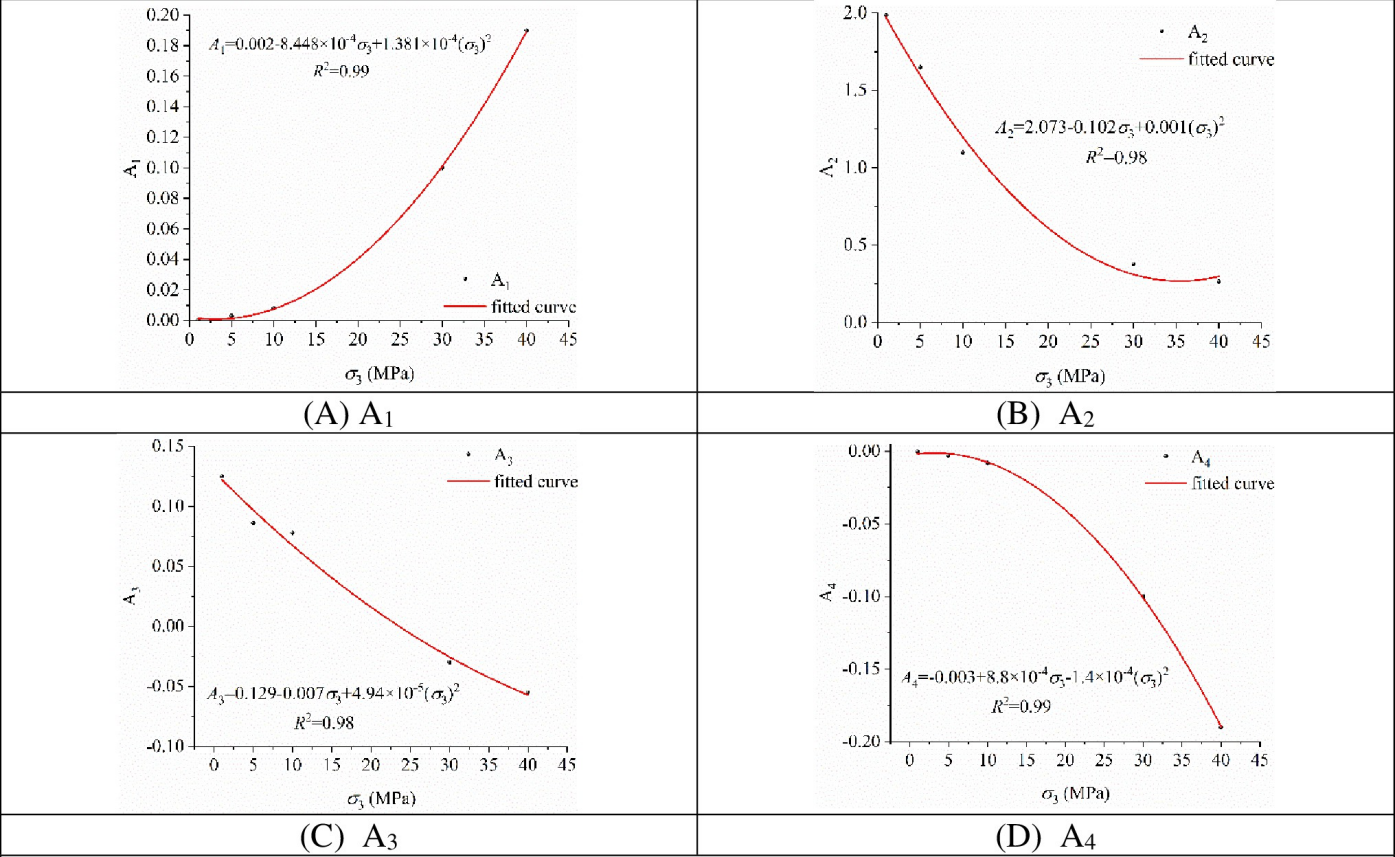
Curves of the coefficients *A*_1_, *A*_2_, *A*_3_ and *A*_4_ versus the confining pressure *σ*_3_.

As shown in Fig 7, the coefficients *A*_1_, *A*_2_, *A*_3_ and *A*_4_ are fitted to the confining pressure *σ*_3_ in Eq (9) to represent the energy dissipation degree at different stress states in the surrounding rock. Eq (10) is the fitting equation.

$$A_{i}=l_{i}+m_{i}\sigma_{3}+n_{i}\sigma_{3}{}^{2},i=1,2,3,$$ (10)

where *l*_*i*_, *m*_*i*_ and *n*_*i*_ are the relevant fitting coefficients, and their values are shown in Table 3.

**Table 3:** The relevant fitting coefficients.

*l*_1_	*m*_1_	*n*_1_	*l*_2_	*m*_2_	*n*_2_	*l*_3_	*m*_3_	*n*_3_	*l*_4_	*m*_4_	*n*_4_
0.002	-8.4×10^−4^	1.4×10^−4^	2.073	-0.102	0.001	0.129	-0.007	4.9×10^−5^	-0.003	8.8×10^−4^	-1.4×10^−4^

Fig 7 shows that the correlation coefficients *R*^2^ all reach above 0.98, which indicates that the coefficients *A*_1_, *A*_2_, *A*_3_ and *A*_4_ all have a clear parabolic-type relationship with the confining pressure *σ*_3_.

Eqs (9) and (10) are empirical formulas for the criterion for dividing the surrounding rock EDZs within the underground cavities based on the energy dissipation degree *R*_*Ud*,_ derived from the numerical analysis of the results of conventional triaxial tests, which take into account both the effects of the surrounding pressure and strain, and they can be used to calculate the value of *R*_*Ud*_ during rock damage at any position in the surrounding rock. Their formats are simple and can therefore be easily applied in engineering.

In practical engineering applications for a specific project, only a set of conventional triaxial tests needs to be carried out, and the empirical formulas for the criterion can be obtained by simple data processing according to the method provided in this paper. In particular, during the numerical simulation calculation, the stress‒strain state of any unit in the model is known, and then the value of the energy dissipation degree *R*_*Ud*_ of the unit can be calculated by Eq (9) and Eq (10). The dissipation energy in the numerical simulation can be calculated by Eq (8) and *Up d* = -0.044+0.015*σ*_3_ (Fig 4(F)). The change in the surrounding rock’s *R*_*Ud*_ can be acquired, and the surrounding rock’s damage degree and range can be divided through quantitative analysis of *R*_*Ud*_, which can provide reference advice on the design of support measures and then analyze and evaluate the surrounding rock’s overall stability.

## 4. Analysis of EDZs in the surrounding rock mass of large underground caverns

### 4.1 EDZs in the surrounding rock mass of underground caverns based on *R*_*Ud*_

**Fig 8:**
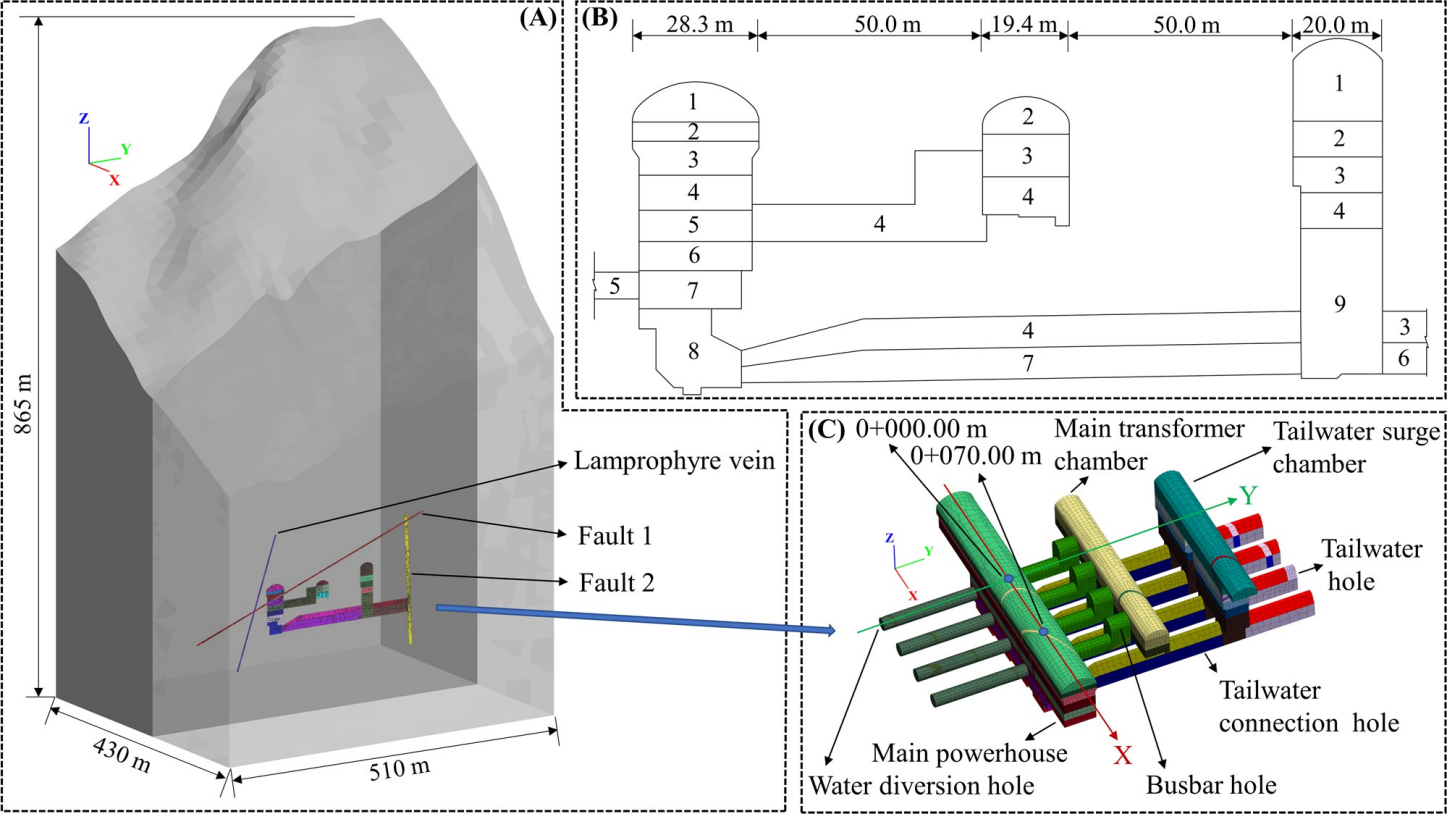
(A) Overall model and calculation scope, (B) excavation sequence, and (C) 3D model of the underground caverns of Shuangjiangkou Hydropower Station.

The calculation object of this numerical simulation model is the left bank mountain where the underground workshop of Shuangjiangkou Hydropower Station is located, as shown in Fig 8(A). The length of the X-axis direction of the model is 430 m (0–180.00~0+250.00 m) in total, the length of the Y-axis is 510 m (0–190.00~0+320.00 m), and the length of the Z-axis is 865 m (2100.00~2965.00 m). The focus of the Z-axis is vertically upward, and the elevation at the bottom is 2100.00 m, with the top extending to the surface of the mountain. The underground caverns of the Shuangjiangkou Hydropower Station include the main powerhouse, main transformer chamber and tailwater surge chamber (whose widest parts are 28.3 m, 19.4 m and 20.0 m), as well as other caverns. There are also three defects distributed in the area, including Fault 1, Fault 2 and a lamprophyre vein. As shown in Fig 8(B), the excavation of the underground workshop caverns is carried out in 9 stages, and the site construction is currently excavated to the 4th stage. The 3D model of the underground caverns is shown in Fig 8(C).

The software used for the numerical simulation in this paper is FLAC^3D^, and the yield criterion is the Morh-Coulomb criterion built into the software. The deformation modulus *E*_*d*_, bulk density *γ*, Poisson’s ratio *μ*, cohesion *c* and internal friction coefficient *f* for numerical simulations are determined by the designer of the project, as listed in Table 4.

**Table 4:** The parameters for numerical simulations of the rock mass of the Shuangjiangkou Hydropower Station.

Type of rock mass	Parameters				
	*E*_*d*_(GPa)	*γ*(kN/m^3^)	*μ*	*c*(MPa)	*f*
Unweathered	12.5	26.0	0.30	1.55	1.25
Weakly weathered	7.0	25.5	0.32	0.90	0.95
Strongly weathered	3.0	23.5	0.35	0.40	0.70
Weak structure plane	0.3	18.0	0.38	0.03	0.40

The inversion of the in situ stress field based on the measured in situ stress indicates that the in situ stress field in the engineering region is mainly composed of tectonic stress and gravitational pressure, and its distribution is influenced by the tectonic stress, geological structure, and local topography [57]. From the top to the bottom of the surface, *σ*_*x*_, *σ*_*y*_ and *σ*_*z*_ increase with depth.

**Fig 9:**
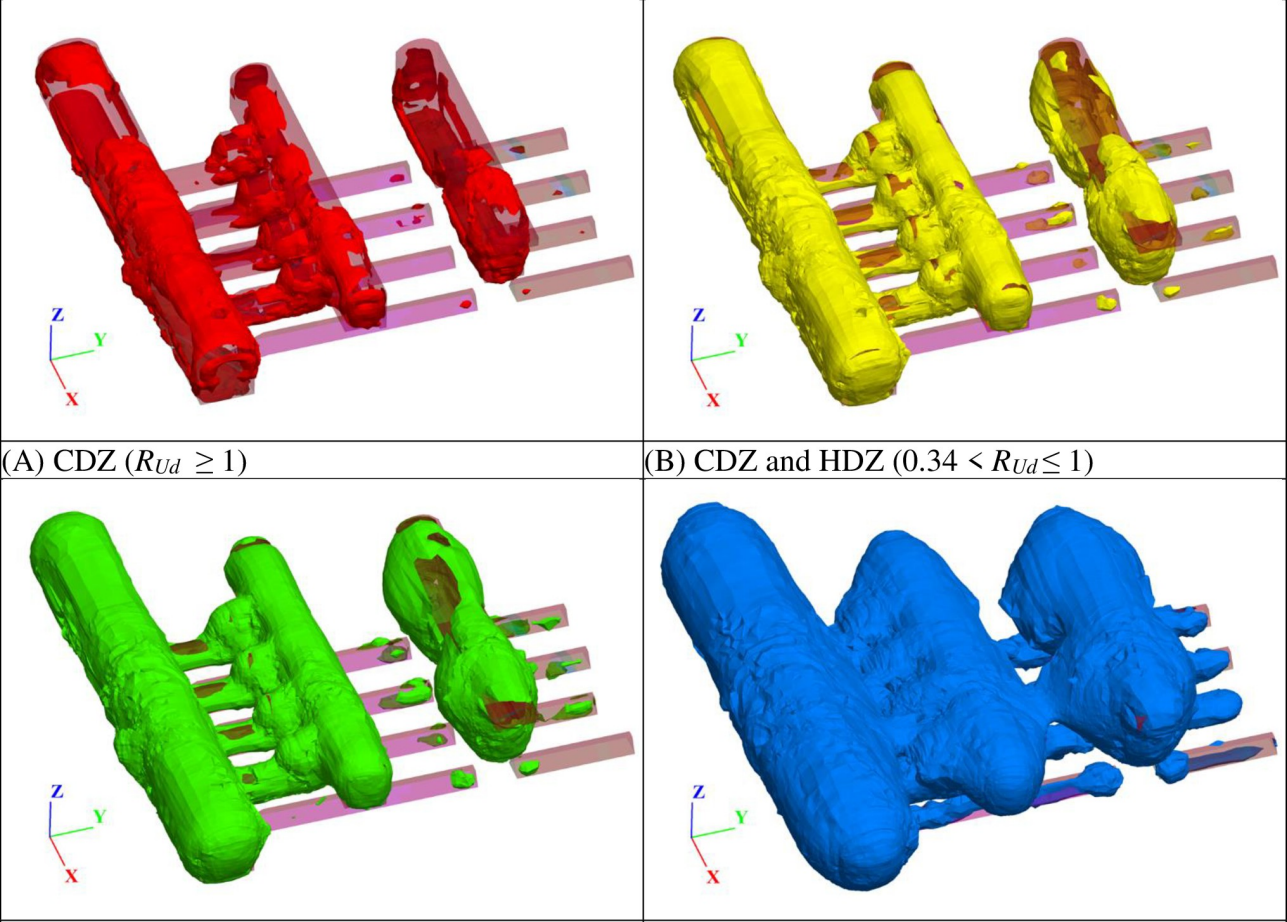
Characteristics of EDZs in surrounding rock of the underground caverns of Shuangjiangkou Hydropower Station.

After the fourth stage of excavation is completed, the minimum principal stress and maximum principal strain of all units in the model can be obtained and brought into Eqs (9) and (10) to derive the value of *R*_*Ud*_. Based on the four thresholds of *R*_*Ud*_ in Table 1, the distribution ranges of the CDZ, HDZ, MDZ and EIZ of the underground caverns can be derived, as shown in Fig 9. The CDZ is mainly distributed in the area of 0.5~3 m around both the cavern and the connection area of the cavern. The HDZ is more widely distributed on the basis of the CDZ when the depth is increased to approximately 1~7 m, and the increase is more obvious in the sidewalls of cavities and the connection area between cavities. The MDZ is increased to approximately 1~12 m on the basis of the HDZ, especially the middle of the cavity sidewall, and the connection area between cavities is increased. The EIZ forms a large interconnected area at the periphery of the MDZ, and it is evident that the depth of the impact of the excavation on the surrounding rock is very large, even greater than 30 m at the deepest point.

### 4.2 EDZs of the surrounding rock mass of underground caverns based on wave velocity

The propagation velocity of elasticity waves varies in different media, and elasticity wave testing techniques have been widely utilized to study EDZs [24, 26]. The rock integrity index (*K*_*v*_) is usually defined as the square of the ratio of the elastic longitudinal velocity of the rock mass (*v*_*p*_) to the elastic longitudinal velocity of the intact rock (*v*_*pr*_), as shown in Eq (11). The higher the wave velocity of the longitudinal waves in the rock mass, the higher the value of *K*_*v*_, indicating the better quality of the rock. *D* is defined as the degree of damage to the rock mass, and the formula is shown in Eq (12); the higher the value of *D* is, the higher the degree of damage to the rock mass.


$$K_{v}={\left(\frac{v_{p}}{v_{pr}}\right)}^{2}$$ (11)

$$D=1-K_{v}$$ (12)

**Fig 10:**
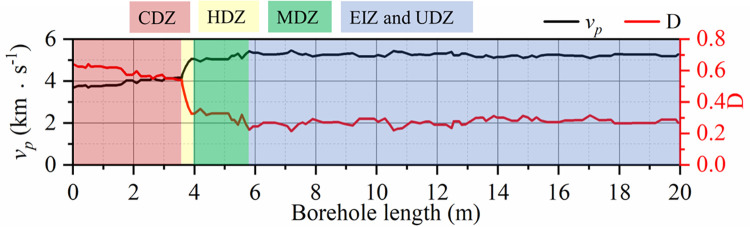
Method of classifying the EDZs of surrounding rock mass according to the variation of wave velocity.

According to the variation curve of *v*_*p*_ with borehole length, the EDZs of the surrounding rock can be divided into several zones, such as the CDZ, HDZ, MDZ, EIZ and UDZ, as shown in Fig 10. In the CDZ, *v*_*p*_ is lower and becomes larger slowly with the borehole length, and *D* decreases slowly. The rock mass in the area is severely damaged and ruptured, and macroscopic fractures are developed. The rock mass loses bearing capacity or has very poor bearing capacity. The deformation of the surrounding rock is serious, and even rock explosion, flake gang, and crumbling occur. The *v*_*p*_ is still low in the HDZ, but it increases rapidly with the borehole length, while *D* decreases rapidly. The rock damage and rupture in the area is more serious, the macroscopic fractures are more developed, and the bearing capacity of the rock is poor. In the MDZ, *v*_*p*_ slowly increases to a peak with the borehole length, and *D* slowly decreases to a very low value. The rock mass in the area has a certain degree of damage rupture, and the majority of the fissures are not penetrated, while the rock has a certain bearing capacity. The *v*_*p*_ and *D* variations in the EIZ and UDZ are not obvious and are difficult to use as a basis for the division of EDZs, so the EIZ and UDZ are unified into one region when the EDZs are divided based only on the wave velocity.

**Fig 11:**
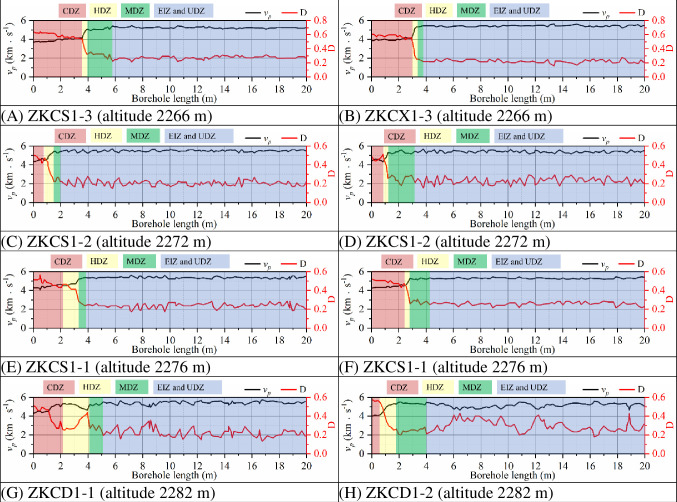
EDZs in the surrounding rock in the 0+070.00 m section of the main powerhouse with the depth of borehole.

**Fig 12:**
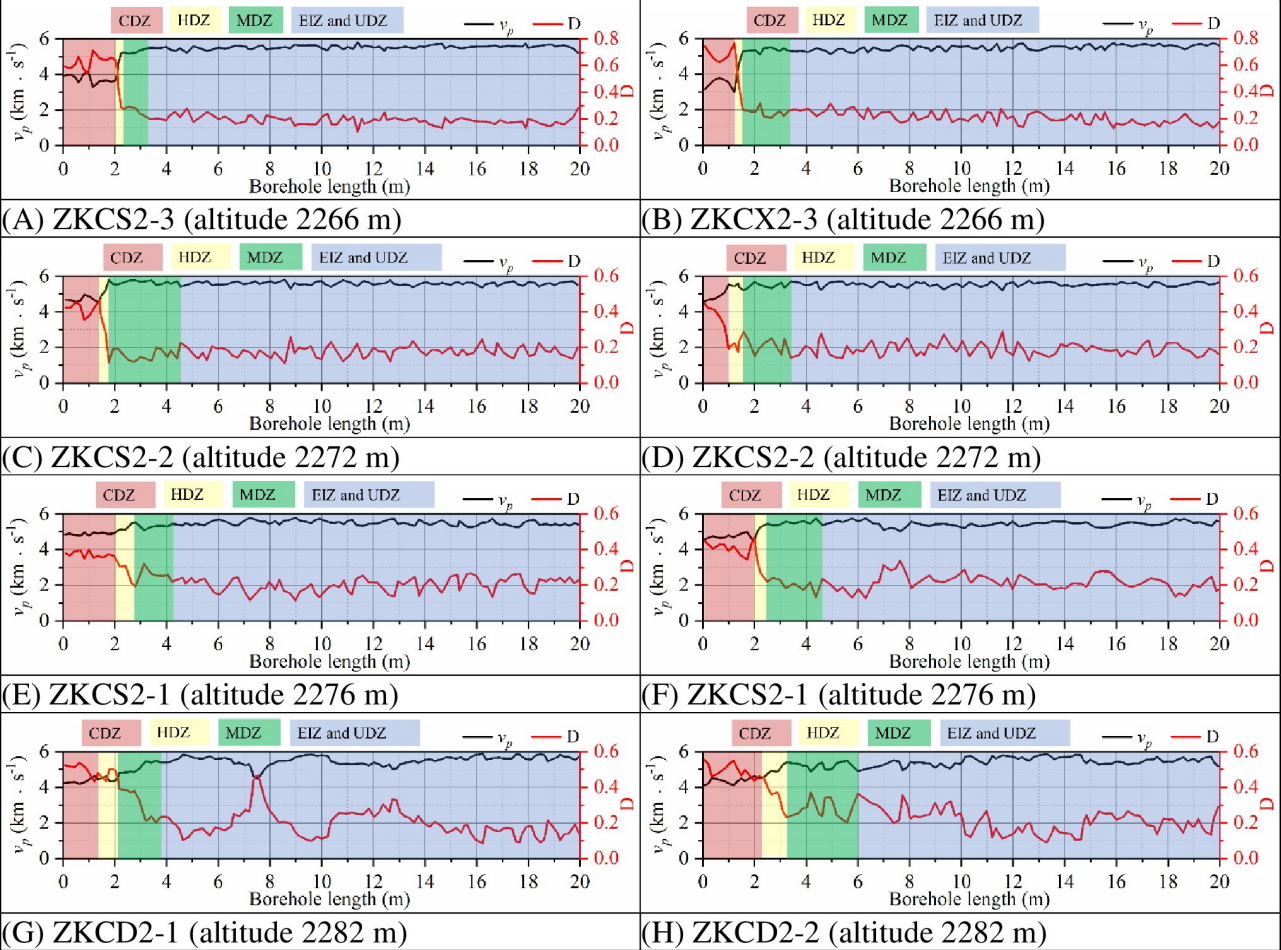
EDZs in the surrounding rock in the 0+100.00 m section of the main powerhouse with the depth of borehole.

After the completion of the excavation of the fourth stage of the underground caverns of Shuangjiangkou Hydropower Station, eight wave velocity test boreholes were arranged in the 0+070.00 m section and 0+100.00 m section for the main powerhouse respectively. Based on the measured wave velocity test data, the EDZ distribution characteristics of the surrounding rock in the 0+070.00 m section and 0+100.00 m section of the underground caverns can be obtained as shown in Figs 11 and 12.

The distribution characteristics of the EDZs based on the elastic wave tests show that the depth variation range of the CDZ of the underground caverns at the 0+070.00 m section of the surrounding rock is 0.6~3.5 m, the depth variation range of the HDZ is 1.8~3.9 m, and the depth variation range of the MDZ is 3.7~5.1 m. The depth variation range of the CDZ of the underground caverns at the 0+100.00 m section of the surrounding rock is 1.0~2.3 m, the depth variation range of the HDZ is 1.5~3.3 m, and the depth variation range of the MDZ is 3.3~6.0 m. The wave velocity in the EIZ region and the UDZ region cannot be accurately determined with the variation in drilling depth, so the EIZ and UDZ can be combined into one region. Based on the analysis of the variation characteristics of wave velocity with drilling depth, the CDZ, HDZ and MDZ can be used as the key areas for support.

### 4.3 Distribution characteristics of the EDZs of the surrounding rock mass of underground caverns

**Fig 13:**
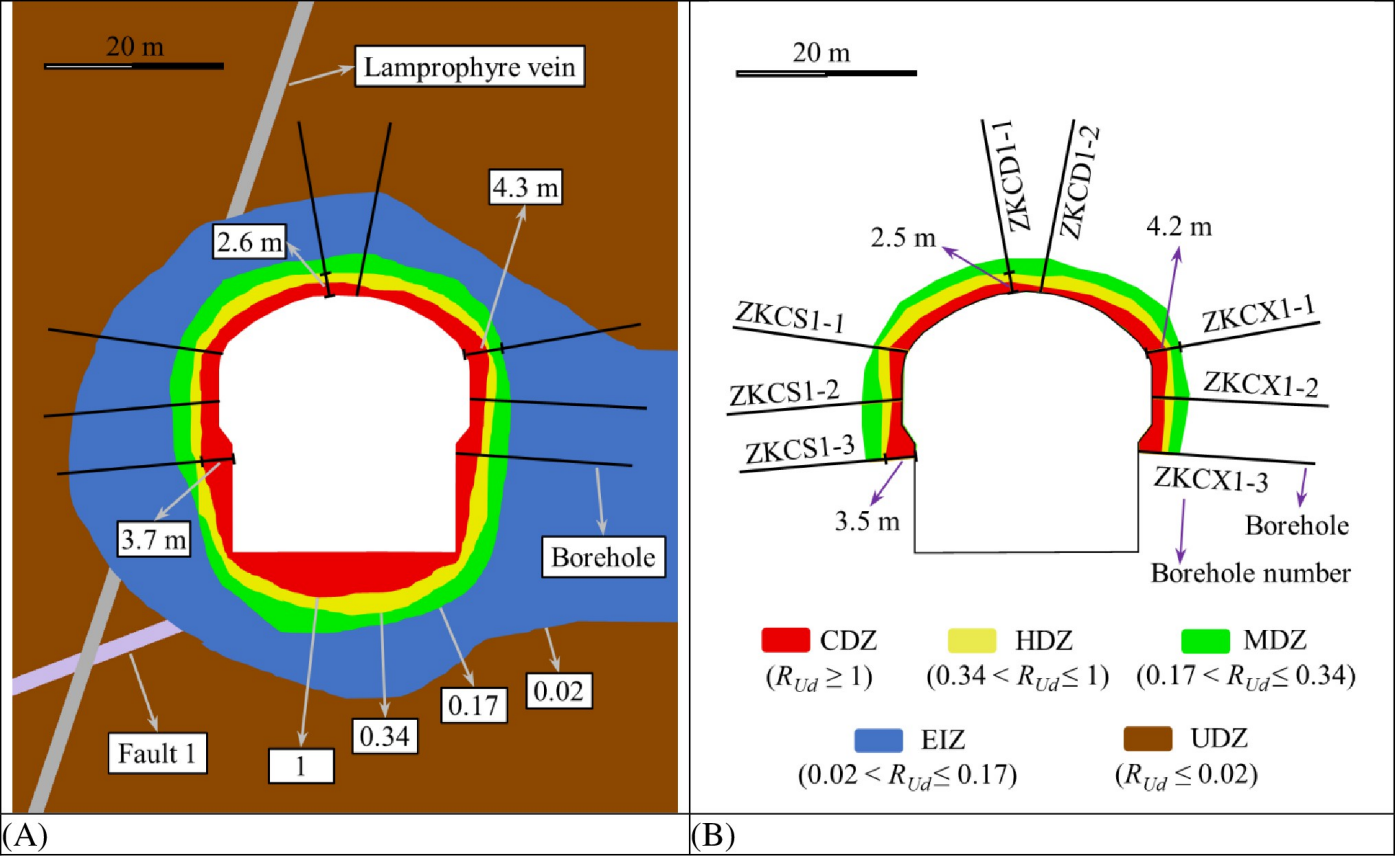
Distribution characteristics of the EDZs of surrounding rock in 0+070.00 m section of the main powerhouse: (A) based on *R*_*Ud*_ thresholds and (B) based on wave velocity.

Fig 13 and Table 5 shows the distribution characteristics of EDZs of the surrounding rock in 0+070.00 m section of the main powerhouse based on *R*_*Ud*_ thresholds and based on wave velocity. In the borehole ZKCD1-1 in the top arch area, the distances from the borehole entrance to the boundaries of the CDZ, HDZ and MDZ obtained with the *R*_*Ud*_-based method are 1.5 m, 2.6 m and 4.1 m, respectively, while the distances obtained with the wave velocity-based method are 1.2 m, 2.5 m and 4.1 m, respectively. In the borehole ZKCX1-1 in the arch shoulder area of the downstream section, the distances from the borehole entrance to the boundaries of the CDZ, HDZ and MDZ obtained with the *R*_*Ud*_-based method are 2.2 m, 2.8 m and 4.3 m, respectively, while the distances obtained with the wave velocity-based method are 2.4 m, 2.7 m and 4.2 m, respectively. In the borehole ZKCS1-3 in the wall area of the upstream section, the distances from the borehole entrance to the boundaries of the CDZ, HDZ and MDZ obtained with the *R*_*Ud*_-based method are 3.7 m, 4.3 m and 6.3 m, respectively, while the distances obtained with the wave velocity-based method are 3.5 m, 3.9 m and 5.1 m, respectively.

**Table 5:** Distance from the EDZ boundary to the borehole entrance in the 0+070.00 m section and 0+100.00 m section of the main powerhouse based on *R*_*Ud*_ thresholds and wave velocity.

Section	Borehole number	Borehole entrance elevation (m)	Distance from EDZs’ boundary to the borehole entrance (m)						Relative error (%)
			Based on *R*_*Ud*_ thresholds			Based on wave velocity			
			CDZ	HDZ	MDZ	CDZ	HDZ	MDZ	
0+070.00 m	ZKCS1-3	2266	3.7	4.3	6.3	3.5	3.9	5.1	17.51
	ZKCX1-3	2266	2.8	3.7	5.8	2.9	3.4	3.7	36.60
	ZKCS1-2	2272	1.8	3.2	5.5	1.0	2.5	4.8	23.13
	ZKCX1-2	2272	1.7	2.9	4.7	1.2	2.5	4.1	17.73
	ZKCS1-1	2276	2.1	3.2	5.0	2.2	3.3	3.8	22.00
	ZKCX1-1	2276	2.2	2.8	4.3	2.4	2.7	4.2	4.42
	ZKCD1-1	2282	1.5	2.6	4.1	1.2	2.5	4.1	6.39
	ZKCD1-2	2282	1.1	2.4	5.0	0.6	1.8	3.8	33.71
0+100.00 m	ZKCS2-3	2266	2.1	3.0	4.1	2.0	2.3	3.3	23.77
	ZKCX2-3	2266	1.8	2.3	4.4	1.2	1.5	3.4	36.21
	ZKCS2-2	2272	2.0	2.8	4.4	1.4	1.8	4.6	23.05
	ZKCX2-2	2272	2.0	2.8	4.1	1.0	1.6	3.5	42.08
	ZKCS2-1	2276	1.6	2.8	4.1	2.0	2.8	4.2	7.59
	ZKCX2-1	2276	1.7	3.1	4.4	1.9	2.4	4.6	13.66
	ZKCD2-1	2282	1.5	2.1	4.4	1.4	2.2	3.8	13.38
	ZKCD2-2	2282	1.7	2.9	4.9	2.3	3.3	6.0	18.21

**Fig 14:**
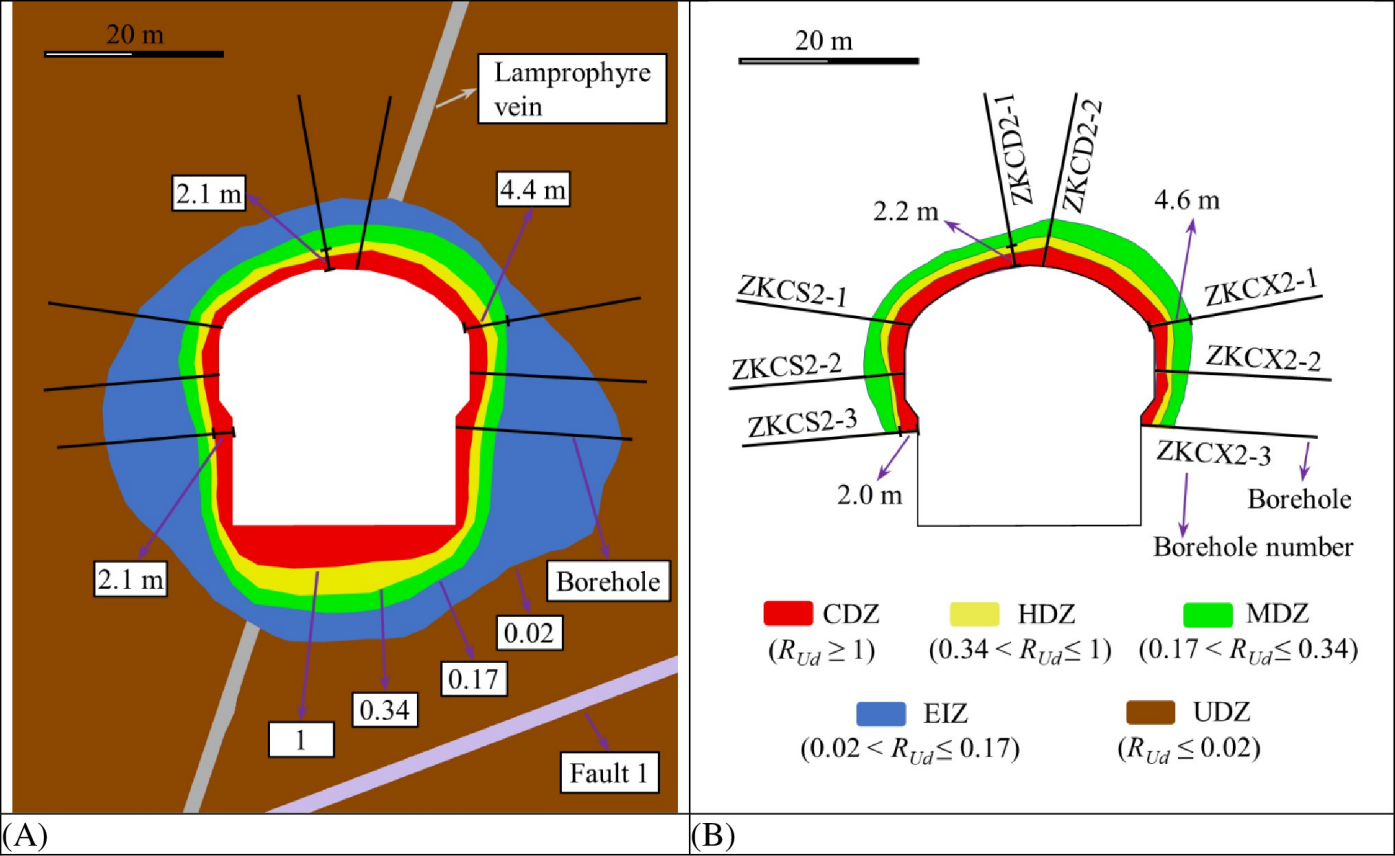
Distribution characteristics of the EDZs of surrounding rock in 0+100.00 m section of the main powerhouse: (A) based on *R*_*Ud*_ thresholds and (B) based on wave velocity.

Fig 14 and Table 5 shows the distribution characteristics of EDZs of the surrounding rock in 0+100.00 m section of the main powerhouse based on *R*_*Ud*_ thresholds and based on wave velocity. In the borehole ZKCD2-1 in the top arch area, the distances from the borehole entrance to the boundaries of the CDZ, HDZ and MDZ obtained with the *R*_*Ud*_-based method are 1.5 m, 2.1 m and 4.4 m, respectively, while the distances obtained with the wave velocity-based method are 1.4 m, 2.2 m and 3.8 m, respectively. In the borehole ZKCX2-1 in the arch shoulder area of the downstream section, the distances from the borehole entrance to the boundaries of the CDZ, HDZ and MDZ obtained with the *R*_*Ud*_-based method are 1.7 m, 3.1 m and 4.4 m, respectively, while the distances obtained with the wave velocity-based method are 1.9 m, 2.4 m and 4.6 m, respectively. In the borehole ZKCS2-3 in the wall area of the upstream section, the distances from the borehole entrance to the boundaries of the CDZ, HDZ and MDZ obtained with the *R*_*Ud*_-based method are 2.1 m, 3.0 m and 4.1 m, respectively, while the distances obtained with the wave velocity-based method are 2.0 m, 2.3 m and 3.3 m, respectively.

The distribution characteristics of the EDZs of the surrounding rock of the underground caverns classified by *R*_*Ud*_-based and wave velocity-based tests show that the depths of the EDZs of the surrounding rock at the cavern sidewalls are greater than those of the top arch, the depths of the EDZs in the middle of the high sidewalls are significantly greater than that of the remaining parts, and the depths of the EDZs in the area affected by Fault 1 are greater than those in the unaffected area. The variation patterns of the EDZs of the surrounding rock classified based on wave velocity tests in the three major caverns in the 0+070.00 m section are generally similar to those based on *R*_*Ud*_. The distribution range of EDZs based on the wave velocity tests is generally slightly smaller than that of EDZs based on *R*_*Ud*_, which is related to the unfinished excavation of the caverns and the low number of wave velocity tests. The distribution characteristics of the underground cavern EDZs are obtained by numerical simulation calculations and in situ elastic wave tests, and the results are basically the same. However, the *R*_*Ud*_-based method for classifying the surrounding rock’s EDZs has the obvious advantage of being able to probe the boundaries of the surrounding rock’s undamaged zone (UDZ) more explicitly, while the method based on wave velocity testing is not sufficiently explicit.

## 5. Discussion

The proposed energy calculation parameter *R*_*Ud*_ represents the degree of dissipation energy change during the damage of the rock, yet there are some other published energy indexes for rock, such as the Local Energy Release Rate (*LERR*) which refers to the sudden release of energy stored in the rock mass per volume when the strain energy concentrated in a local area of the rock is larger than its limiting capacity during excavation. The formula for *LERR* can be written as [58, 59]:

$$LERR=U_{e\max}-U_{e\min}$$ (13)

where *U*_*e*max_ and *U*_*e*min_ are the peak and trough values of elastic strain energy intensity before and after brittle failure of the element respectively. *LERR* studies the release value of the elastic strain energy of the local surrounding rock after brittle damage, which is applied in numerical simulation calculations to predict the strength of the rock burst and the depth of the burst crater, while *R*_*Ud*_ studies the damage zoning of the surrounding rock from the perspective of dissipation energy. The *R*_*Ud*_-based criterion can divide the EDZ into five zones, including CDZ, HDZ, MDZ, EIZ, and UDZ, while the range of rock bursts studied based on *LERR* is equivalent to the CDZ based on the *R*_*Ud*_-based criterion. In addition, the damage zoning of the surrounding rock based on *R*_*Ud*_ can provide support design advice for the excavation of the surrounding rock, such as the support method, the length of the free section and anchor section of the prestressing anchor, etc.

**Fig 15:**
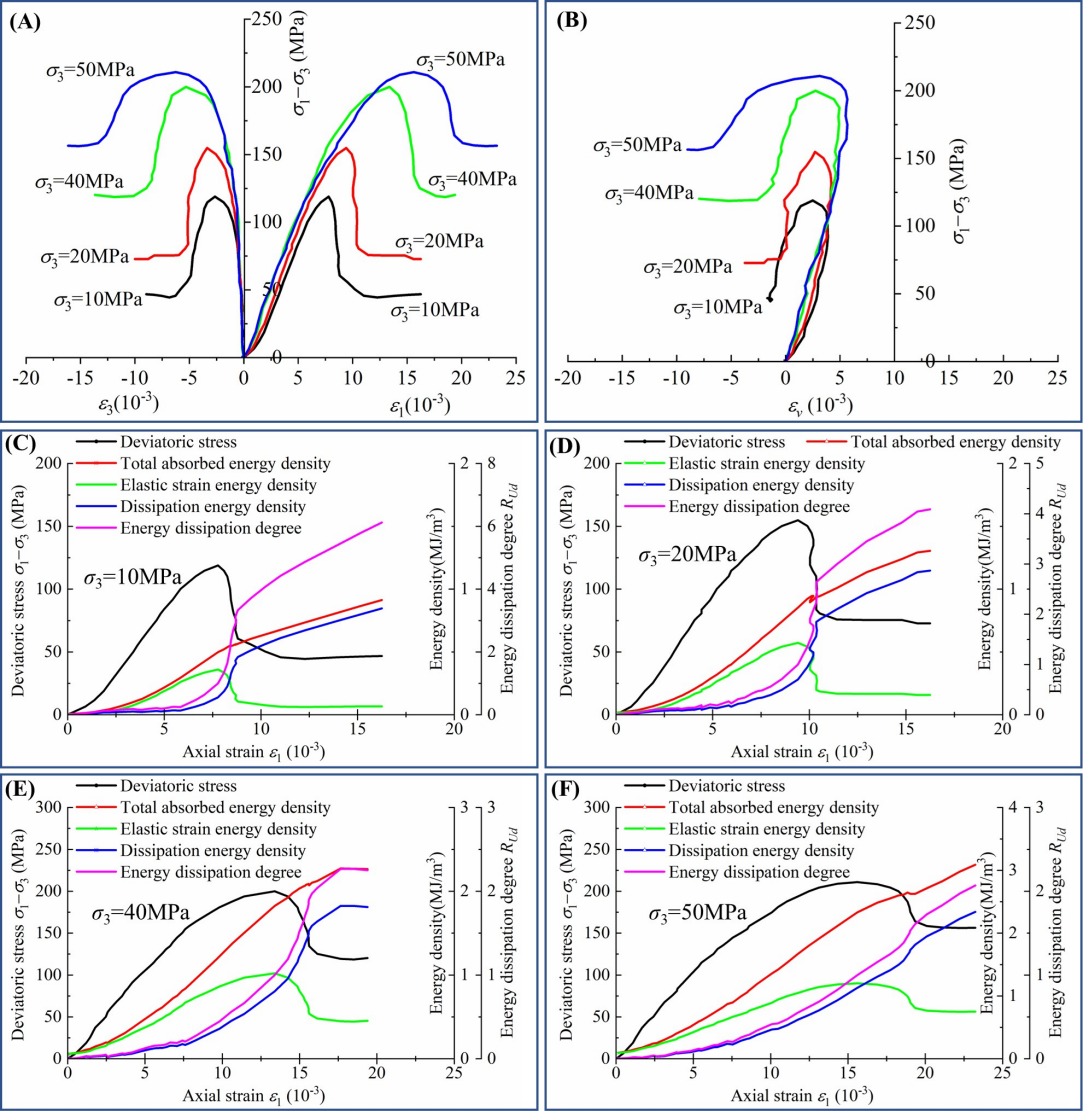
The conventional triaxial test data and associated curves for brittle sandstone [59].

Referring to the conventional triaxial test data for brittle sandstone [59], the threshold values of *R*_*Ud*_ for different confining pressures were analyzed by applying the method provided in this paper and are shown in Fig 15 and Table 6. It is found that the same threshold value of *R*_*Ud*_ for brittle sandstone under different confining pressures varies within an interval that can be approximated as a constant, but is different from that of granite. Therefore, in the application of the method of this paper, the threshold values of *R*_*Ud*_ need to be determined on the basis of experiments for different rocks.

**Table 6:** The threshold values of energy dissipation degree *R*_*Ud*_ for brittle sandstone.

*σ*_3_ (MPa)	*σ*_*cc*_ (MPa)	*R*_*Udc*_		*σ*_*ci*_ (MPa)	*R*_*Udi*_		*σ*_*cd*_ (MPa)	*R*_*Udd*_		*σ*_*p*_ (MPa)	*R*_*Udp*_
10	10.2	0.10	0.08	46.7	0.20	0.16	98.5	0.26	0.24	118.9	1
20	20.4	0.09		60.2	0.19		114.4	0.25		154.9	
40	23.3	0.06		71.3	0.12		143.2	0.20		199.9	
50	27.5	0.04		80.2	0.13		151.5	0.22		210.9	

The criterion proposed in this paper is based on the analysis of conventional triaxial loading test data of intact granite, without considering the influence of unloading effect and existing fissures, which are important factors affecting the stability of underground cavern excavation [60–62], and we will make corresponding research in related fields in the next step.

## 6. Conclusion

The paper studied the energy dissipation mechanism and energy evolution characteristics during conventional triaxial tests of granite in Shuangjiangkou Hydropower Station, proposed the criterion for dividing the EDZs of the surrounding rock mass of underground caverns based on the energy dissipation degree *R*_*Ud*_, analyzed the stability of the surrounding rock in the underground cavern of Shuangjiangkou Hydropower Station, came to the following conclusions.

1. The four stress thresholds (*σ*_*cc*_, *σ*_*ci*_, *σ*_*cd*_ and *σ*_*p*_) correspond to the four *R*_*Ud*_ thresholds (*R*_*Udc*_, *R*_*Udi*_, *R*_*Udd*_ and *R*_*Udp*_) on the energy dissipation degree-strain curve. The stress threshold at different confining pressures becomes larger with increasing confining pressure, while the corresponding threshold of energy dissipation degree *R*_*Ud*_ varies in a smaller interval at different confining pressures. The same threshold has good consistency under different confining pressures, and *R*_*Udc*_ can be taken as 0.02, *R*_*Udi*_ as 0.17, *R*_*Udd*_ as 0.34, while *R*_*Udp*_ is 1. The criterion uses four energy dissipation degree thresholds (*R*_*Udc*_, *R*_*Udi*_, *R*_*Udd*_ and *R*_*Udp*_) to classify the surrounding rock mass into five categories: CDZ, HDZ, MDZ, EIZ and UDZ.
2. Based on the criterion, the EDZs of the surrounding rocks of the underground cavern group of the Shuangjiangkou Hydropower Station are analyzed. The CDZ is mainly distributed in the area of 0.5~3 m around both the cavern and the connection area of the cavern. The HDZ is more widely distributed on the basis of the CDZ when the depth is increased to approximately 1~7 m, and the increase is more obvious in the sidewalls of cavities and the connection area between cavities. The MDZ is increased to approximately 1~12 m on the basis of the HDZ, especially the middle of the cavity sidewall, and the connection area between cavities is increased. The EIZ forms a large interconnected area at the periphery of the MDZ, and it is evident that the depth of the impact of the excavation on the surrounding rock is very large, even greater than 30 m at the deepest point.
3. The distribution characteristics of the EDZs of the underground caverns of the Shuangjiangkou Hydropower Station at the 0+070.00 m section and the 0+100.00 m section of the surrounding rock are obtained based on in situ wave velocity tests and are compared with those based on *R*_*Ud*_. The *R*_*Ud*_-based method for classifying the EDZs of the surrounding rock has the obvious advantage of being able to probe the boundaries of the UDZ more explicitly, while the method based on wave velocity testing is not sufficiently explicit. Generally, the CDZ, HDZ and MDZ should be used as the focus areas for support.

## List of symbols

γ: bulk density (kN/m^3^)
μ: Poisson’s ratio
E: elastic modulus (GPa)
*E*_*d*_: deformation modulus (GPa)
c: cohesion (MPa)
f: internal friction coefficient
*σ*_1_: axial stress
*ε*_1_: axial strain
*σ*_3_: lateral stress
*ε*_3_: lateral strain
*σ*_*cc*_: crack closure stress
*σ*_*ci*_: crack initiation stress
*σ*_*cd*_: damage stress
*σ*_*p*_: peak stress
U: total absorbed energy density
*U*_*l*_: freezing energy density
*U*_*e*_: elastic strain energy density
*U*_*d*_: dissipated energy density
Up d: dissipated energy at the peak stress *σ*_*p*_
*U*_0_: energy density caused by hydrostatic loading
*U*_1_: energy density owing to axial compression by *σ*_1_ after hydrostatic pressure
*U*_3_: energy density owing to lateral compression by *σ*_3_ after hydrostatic pressure
*R*_*Ud*_: energy dissipation degree
*R*_*Udc*_: the *R*_*Ud*_ threshold corresponding to *σ*_*cc*_
*R*_*Udi*_: the *R*_*Ud*_ threshold corresponding to *σ*_*ci*_
*R*_*Udd*_: the *R*_*Ud*_ threshold corresponding to *σ*_*cd*_
*R*_*Udp*_: the *R*_*Ud*_ threshold corresponding to *σ*_*p*_
*v*_*p*_: elastic longitudinal velocity of the rock mass
*v*_*pr*_: elastic longitudinal velocity of the intact rock
*K*_*v*_: rock integrity index
D: degree of damage to the rock mass
